# Acute Intoxication After Baclofen Administration: A Review of the Literature and Methodological Proposals

**DOI:** 10.3390/toxics13110999

**Published:** 2025-11-20

**Authors:** Giuseppe Davide Albano, Mauro Midiri, Péter Attila Gergely, Tamás Gergő Harsányi, Kálmán Racz, Alessandra Matilde Nivoli, Roberto Buscemi, Stefania Zerbo, Antonina Argo, Claudia Trignano

**Affiliations:** 1 Institute of Legal Medicine, Department of Health Promotion, Mother and Child Care, Internal Medicine and Medical Specialties, University of Palermo, 90133 Palermo, Italyantonella.argo@unipa.it (A.A.); 2 Department of Forensic Medicine, Faculty of Medicine, University of Debrecen, 4032 Debrecen, Hungary; 3 Department of Medicine, Surgery and Pharmacy, University of Sassari, 07100 Sassari, Italy; 4 Department of Biomedical Sciences, University of Sassari, 07100 Sassari, Italy

**Keywords:** baclofen, overdose, intrathecal therapy, forensic toxicology, postmortem interpretation

## Abstract

Baclofen intoxication, once rare, is now increasingly observed in clinical and forensic settings due to its expanding medical and off-label use. However, baclofen is not routinely included in standard postmortem toxicological panels and is usually tested only when explicitly requested. This selective approach, together with the lack of validated cut-offs and standardized interpretative protocols, complicates both clinical management and postmortem evaluation. A systematic review of the literature published between July 2005 and July 2025 was conducted according to PRISMA guidelines, including fatal and non-fatal baclofen intoxications with quantitative toxicological data. Analytical methods, biological matrices, concentration ranges, and clinical outcomes were compared to identify recurring patterns and interpretative gaps. A fatal intrathecal overdose case was also analyzed as a paradigmatic example of diagnostic and methodological challenges. In thirteen studies meeting inclusion criteria and comprising over 300 cases, reported blood concentrations ranged from 0.04 to 110 mg/L, with overlapping values between survivors and fatalities. The analysis revealed marked heterogeneity in matrices and methods, and a poor correlation between concentration and clinical severity, limiting the reliability of toxicological interpretation in both clinical and postmortem settings. Baclofen intoxication illustrates the challenges of interpreting toxicological data without harmonized analytical criteria and highlights the need for standardized procedures and shared reference databases to improve diagnostic and medico-legal accuracy.

## 1. Introduction

Baclofen is a lipophilic derivative of γ-aminobutyric acid (GABA) acting as a selective agonist of GABA-B receptors. Since its introduction in the 1970s, it has been widely prescribed for the management of spasticity associated with multiple sclerosis, cerebral palsy, and spinal cord injuries [1,2,3,4]. More recently, its clinical use has expanded, particularly in the treatment of alcohol use disorder (AUD), often at high or off-label doses, increasing its availability and the risk of intoxication [5,6,7].

Although considered safe at therapeutic concentrations (80–400 ng/mL in plasma) [8,9], baclofen intoxication may cause a broad spectrum of manifestations, ranging from confusion and hallucinations to seizures, coma, and respiratory depression [10,11,12,13]. Severe intoxications may mimic brain death, with absent brainstem reflexes and burst-suppression EEG patterns [14,15,16,17]. Both oral and intrathecal overdoses have been reported, sometimes in relation to therapeutic errors or pump malfunction [18,19].

Toxic and lethal concentrations of baclofen are still debated, due to significant interindividual variability and frequent co-ingestion of other psychoactive substances. Fatalities have been documented at postmortem blood concentrations above 30 µg/mL [20], while survival has occurred at higher levels [21,22]. Large retrospective series from Australia [23] and France [24] confirmed the increasing burden of baclofen-related intoxications, particularly in intentional self-poisonings and in patients with comorbidities.

Despite this, baclofen is not routinely included in toxicological screening panels [25,26,27], and no standardized forensic protocols exist for interpreting concentrations or for guiding clinical and postmortem investigations [28,29,30], which may lead to under-detection in unexpected deaths unless specifically requested by the pathologist or the investigative authorities.

Starting from the observation of a fatal intoxication following intrathecal baclofen administration, we decided to systematically review the literature on fatal and non-fatal cases over the last 20 years, to identify recurrent patterns, highlight knowledge gaps, and propose methodological recommendations for clinical, toxicological, and forensic practice.

## 2. Materials and Methods

### 2.1. Protocol

A systematic literature search was conducted by two authors (C.T. and G.D.A) independently for studies published between July 2005 and July 2025. The protocol for this study followed the Preferred Reporting Items for Systematic Reviews and Meta-Analyses (PRISMA) guidelines using the methodology described in the Cochrane Collaboration Handbook on Systematic Reviews of Health Promotion and Public Health Program.

### 2.2. Data Sources and Search Strategy

The records were retrieved using different search engines (PubMed and SCOPUS). For the search, MeSH terms and free-text words were combined using Boolean operators as follows: BACLOFEN AND (Death), AND (Fatal Intoxication), AND (Poisoning), AND (Drug Abuse), AND (Misuse), AND (Overdose). The research was completed in July 2025. Reference lists of included papers were hand-searched to identify additional eligible studies.

### 2.3. Inclusion and Exclusion Criteria

We included original reports and case series with data regarding fatal and nonfatal baclofen intoxications. Only studies published in the last twenty years (≥2005) and written in English were considered. To be eligible, the full text had to be available, and the study had to present original data concerning cases of baclofen intoxication confirmed by toxicological investigations performed with a described analytical methodology. Moreover, inclusion required the presence of analytical or toxicological data, including quantitative baclofen concentrations, with a clear indication of the biological matrix and measurement units.

We excluded reviews, systematic reviews, and meta-analyses; conference materials, such as posters, abstracts; non-English articles; in vivo/in vitro studies; and reports lacking toxicological analyses or without quantitative baclofen concentrations or without explicit matrix/unit specification.

### 2.4. Study Selection and Data Collection Process

**Figure 1:**
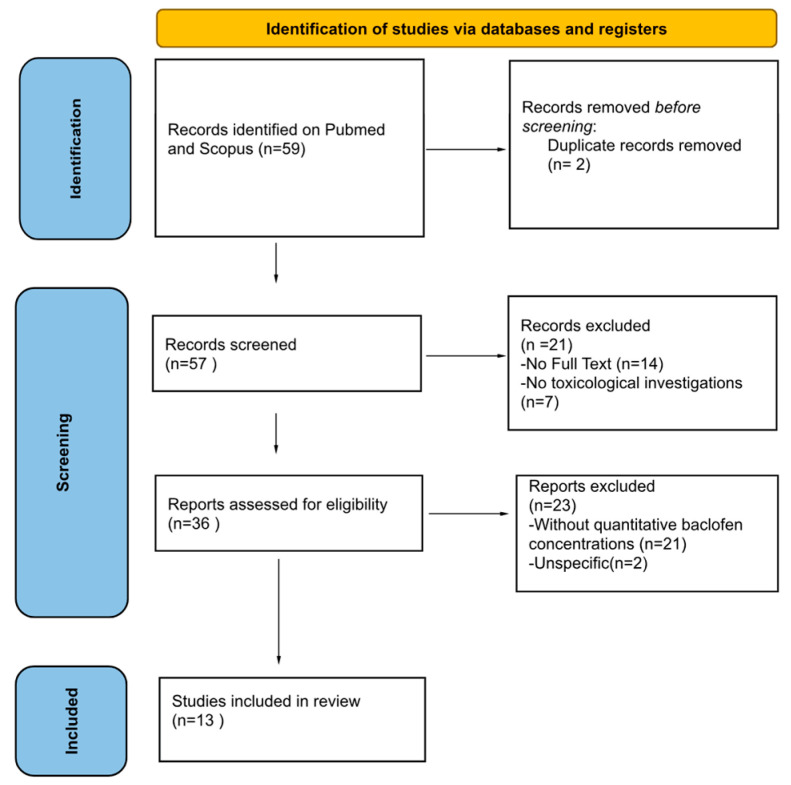
The research methodology is illustrated. Thirteen studies were included in the review according to the established inclusion and exclusion criteria.

Initially, articles were screened on their titles and abstracts. Subsequently, a full-text evaluation of the selected studies was carried out. The search identified 59 records; two duplicates were removed, and 57 records were screened. Then, 21 records were excluded due to the application of the exclusion criteria. After full-text assessment, 23 additional records were excluded as non-specific to baclofen intoxication with quantitative analytics. The quality of each study was evaluated independently by M.M. and G.D.A. If there was a conflict of opinions regarding the articles, they were submitted to C.T. Finally, 13 articles were included in the current review (Figure 1).

For each study, three authors (C.T., G.D.A., and M.M.) independently extracted the following data using a pre-designed data extraction form in an Excel sheet. Study characteristics (first author, year of publication, study design, and study population), sample characteristics (number of cases, sex distribution, age range or mean/median age, clinical context for non-fatal cases), and toxicological methodology and findings (biological matrices analyzed, analytical techniques applied, baclofen concentrations with units) were collected when available. Key clinical findings were also extracted, including clinical manifestations, complications, predisposing factors such as renal dysfunction or concomitant drug use, therapeutic interventions (e.g., dialysis or hemofiltration), and information related to the route or modality of use.

### 2.5. Case Report

As an explanatory example, we present the case of a 53-year-old Caucasian male with multiple sclerosis and spastic paraparesis on chronic intrathecal baclofen therapy since 2007. In June 2019, he underwent scheduled replacement of the intrathecal pump (Medtronic SynchroMed II, Medtronic, Inc., Minneapolis, MN, USA). The device was refilled with baclofen (1000 µg/mL; daily dose 70 µg). Approximately ten hours after the procedure, the patient was found unresponsive and declared dead. A complete autopsy was performed 72 h after death, with the body stored at +4 °C in a cold storage.

Autopsy revealed massive pulmonary edema, cerebral congestion, and multiorgan vascular stasis, with no traumatic injuries. Histological examination confirmed these findings, showing alveolar flooding consistent with acute pulmonary edema, diffuse vascular congestion of the liver, kidneys, and brain, and myocardial lipomatosis. These features supported acute cardiorespiratory failure as the immediate mechanism of death.

Toxicological analysis of peripheral blood (GC–MS) revealed a baclofen concentration of 51.03 µg/mL, far above the expected levels after intrathecal administration (<5 ng/mL). No other xenobiotics were detected; other matrices were not collected. The cause of death was determined as acute baclofen intoxication due to accidental overdose or device malfunction.

This case is not presented as an isolated report, but rather as an illustrative example of the diagnostic and interpretative challenges that emerged repeatedly in the literature reviewed.

## 3. Results

### 3.1. Characteristics of Articles Included in the Systematic Review

The literature search ultimately yielded 13 studies published between 2006 and 2024 that fulfilled the predefined inclusion criteria. Most of the available evidence consisted of single case reports and small series [11,12,13], which provided in-depth clinical and toxicological descriptions of individual intoxications. These were complemented by two extensive retrospective investigations: a multicenter French study that collected 190 cases of self-poisoning reported to a Poison Control Centre [24], and a national Australian series describing 102 baclofen-related deaths over more than two decades [23]. Additionally, an extensive postmortem screening study of 3750 femoral blood samples from the UK was reported, of which 21 were positive for baclofen [22].

Taken together, these studies covered more than 300 patients with either fatal or non-fatal baclofen intoxication. Earlier publications were generally focused on isolated cases, often with meticulous reporting of analytical results and clinical presentation. In contrast, more recent contributions broadened the perspective by describing larger cohorts, thereby offering epidemiological insights into patterns of misuse and fatal intoxications, particularly in the context of the expanding use of baclofen for alcohol use disorder (AUD) and spasticity management.

### 3.2. Sociodemographic and Circumstantial Data

The age distribution across studies was heterogeneous. In the larger cohorts, the median age was in the fourth to fifth decade of life (around 39–46 years). In contrast, case reports highlighted both very young patients, including adolescents as young as 16 years, and older adults over 70 years. Across the available data, a slight predominance of males (approximately 55%) was observed.

The circumstances of intoxication varied widely. In the Australian series, more than half of the deaths (54.9%) were the result of intentional self-poisoning, typically against a background of psychiatric comorbidities and polysubstance use [23]. Similarly, in the French multicentric cohort, baclofen ingestion was most often deliberate, with many patients having a history of alcohol dependence or mood disorders [24]. Among the non-fatal cases, a different pattern emerged: several were linked to therapeutic mishaps, such as accidental overdosing during off-label high-dose treatment for AUD [31], or complications of intrathecal pump therapy, including refill errors and mechanical malfunctions [18,19]. Recreational use in adolescents and young adults was also reported, often with dramatic clinical presentations, despite relatively modest serum concentrations [11].

Additional risk factors became apparent when comparing across studies. Renal impairment was consistently associated with more severe toxicity, owing to the primarily renal elimination of baclofen [31]. The increasing off-label prescription of high daily doses for AUD also emerged as a contributing factor in severe presentations [24]. Finally, co-ingestions were very common, particularly in fatal cases; in the Australian cohort, 93.8% of deaths involved concomitant drugs, most frequently antidepressants and benzodiazepines [23].

### 3.3. Analytical Toxicology Findings

All studies included in this review provided analytical confirmation of baclofen exposure, although the biological matrices and methods used were heterogeneous. Most commonly, concentrations were determined in peripheral blood or plasma; however, urine, vitreous humor, and cerebrospinal fluid (CSF) were also analyzed in select cases. Techniques ranged from LC–MS/MS and HRMS to HPLC, GC–MS, and immunoassays, reflecting differences in laboratory resources and study aims.

The reported baclofen concentrations showed substantial variability across studies. In fatal intoxications, blood levels ranged from 0.04 to 110 mg/L in the Australian series [23], with individual reports documenting 30.7 µg/mL in postmortem blood [20], 1510 mg/L in a chronic alcohol user with renal dysfunction [31], and up to 2062 µg/mL following intrathecal overdose [18]. Additional case reports described fatal or near-fatal concentrations of 4.3 µg/mL [15] and 2.7 µg/mL [14].

In non-fatal intoxications, markedly lower values were observed. Symptomatic cases were reported with levels as low as 0.08–0.28 µg/mL [22], while severe toxicity occurs at 1.81 mg/L [21] and 4.4 µg/mL [29]. Recreational use was associated with disproportionately high urinary concentrations (64,900 ng/mL) despite moderate plasma levels (420 ng/mL) [11].

Intrathecal overdoses presented a unique challenge. Serum concentrations were often within or close to the therapeutic range. At the same time, CSF values were significantly elevated, reaching up to 2062 µg/mL [18] and 7471 ng/mL [19]. These findings underscore the importance of matrix selection in interpreting baclofen concentrations and reinforce that serum alone may underestimate central exposure in intrathecal scenarios.

Overall, the review demonstrates that the therapeutic range of baclofen in blood (0.08–0.40 µg/mL) provides only limited guidance when evaluating suspected intoxications. The correlation between ingested dose, serum level, and clinical severity was frequently poor, and several cases describe prolonged coma despite concentrations close to the therapeutic range. This discrepancy likely reflects the drug’s pharmacokinetics, including redistribution into the central nervous system.

Interventional data further illustrated the role of extracorporeal techniques. Hemodialysis shortened the elimination half-life from 15.7 to 3.1 h in one severe overdose [32], while continuous venovenous hemofiltration (CVVH) increased clearance by 57%, reducing the half-life to 4.8 h [21]. These indicate that dialysis-based therapies can be effective not only in patients with renal impairment but also in those with severe intoxication and preserved renal function. Beyond the reduction in baclofen elimination half-life, several reports suggest that hemodialysis and other extracorporeal techniques may accelerate neurological recovery and shorten the duration of coma in such cases. The characteristics of the studies included in the review are summarized in Table 1.

## 4. Discussion

The present systematic review of 13 studies, complemented by a fatal intrathecal overdose case, highlights several recurring challenges for clinicians, toxicologists, and forensic practitioners in interpreting baclofen intoxication. The case reflects several of the procedural and interpretative shortcomings observed in the literature, particularly concerning postmortem sampling and toxicological workflow.

### 4.1. Scarcity of Systematic Data

Most of the available evidence is based on isolated case reports or small series, with only a few extensive retrospective studies providing broader epidemiological insights. While case reports offer valuable insights into clinical manifestations and toxicological findings, they do not permit robust generalizations or the establishment of evidence-based thresholds. Only a few retrospective investigations have provided larger-scale insights, such as the multicenter French study reporting 190 cases of self-poisoning [24] and the national Australian series documenting 102 baclofen-related deaths [23]. Similarly, Nahar et al. [22] conducted a systematic postmortem screening of 3750 cases, identifying 21 baclofen-positive samples.

In this regard, a large population-based French cohort including over 165,000 patients treated for alcohol use disorder showed a dose-dependent association between baclofen exposure and the risk of hospitalization and death compared with other anti-craving drugs, underscoring that baclofen-related toxicity may extend beyond sporadic case reports and represent a broader pharmacovigilance issue [33].

The current evidence remains fragmented and heterogeneous, underscoring the urgent need for multicenter toxicological registries and internationally coordinated pharmacovigilance systems that can collect standardized data, enabling more comprehensive and comparable analyses [34,35].

### 4.2. Absence of Reliable Toxicological Cut-Offs

A striking feature of the literature is the wide variability of blood concentrations reported in both fatal and non-fatal cases. This heterogeneity is further compounded by the lack of standardized reporting formats and by inconsistent measurement units across studies, which complicate inter-study comparisons. Fatal outcomes have been documented at 30.7 µg/mL, whereas survival has been reported with concentrations as high as 1.81 mg/L under extracorporeal treatment. Such inconsistency demonstrates the lack of a precise dose–effect correlation. The poor alignment between measured levels and clinical severity reflects the complexity of baclofen pharmacokinetics, including interindividual variability, redistribution into the central nervous system, and the influence of comorbid conditions [36]. For this reason, the assessment of baclofen intoxication should primarily rely on clinical presentation and renal function rather than on absolute blood concentrations, which provide limited information about toxicity severity due to wide interindividual variability and matrix-dependent redistribution.

Interestingly, the correlation between clinical severity and plasma concentration is further complicated by cases showing severe neurological suppression at moderate or even therapeutic levels. McGowan and Betten [37] described a case of complete coma with absent brainstem reflexes and a burst-suppression EEG pattern, mimicking anoxic brain injury, with full recovery after 48 h of supportive care. Similar transient “brain-death-like” presentations were also observed by Miller et al. [38], confirming that baclofen toxicity can reversibly suppress brainstem function without permanent neuronal damage.

These observations highlight the importance of exercising caution before establishing a prognosis in comatose patients with suspected baclofen exposure. Prolonged observation and comprehensive toxicological evaluation are essential to avoid premature prognostic conclusions, particularly when analytical data on baclofen concentrations are unavailable, as in these two cases [37,38]. Consequently, continuous toxicological surveillance and close clinical monitoring remain crucial in managing instances of critical baclofen intoxication [39].

In this regard, the pharmacokinetics of baclofen remain insufficiently characterized, particularly under pathological conditions. Renal dysfunction has been consistently identified as a factor prolonging toxicity due to reduced clearance. At the same time, chronic use of high doses, especially in alcohol use disorder (AUD), may contribute to drug accumulation and unpredictable outcomes [40]. The impact of renal impairment also deserves special attention, as even minimal doses can induce encephalopathy when clearance is compromised. Malak and Barzegar [41] reported a 6-year-old boy with advanced renal failure who developed deep coma and areflexia after receiving only 20 mg of baclofen. This pediatric case highlights that dose adjustments-or complete avoidance-are crucial in patients with renal dysfunction, regardless of age, to prevent unpredictable accumulation and neurotoxicity. Moreover, intrathecal overdoses present additional complexity, as systemic concentrations may remain relatively low despite profound central nervous system toxicity, with serum levels often close to the therapeutic range but cerebrospinal fluid concentrations dramatically elevated, as demonstrated in pump-related malfunctions [18,19]. Although plasma concentrations usually underestimate central exposure, the exceptionally high value observed in our case can be attributed to systemic spread secondary to intrathecal pump malfunction and represents an opposite scenario probably related to postmortem redistribution rather than true ante-mortem pharmacokinetics.

This discrepancy highlights the importance of selecting the appropriate biological matrix in both clinical and forensic interpretations, as plasma alone may underestimate central exposure. Furthermore, a thorough assessment of the patient’s clinical history is crucial for accurately contextualizing toxicological findings. This observation, together with the lack of standardized and comparable toxicological data in the literature, underscores the challenges related to the interpretation of baclofen concentrations in different biological matrices in life and post-mortem. Likewise, the standardization of concentration units and analytical reporting formats across studies would greatly facilitate data comparison and improve the interpretative reliability of toxicological results. This issue is very important in the field of forensic toxicology, where reproducibility and reliability of the findings and the methods are crucial to support evidence-based proofs [42,43,44,45,46].

### 4.3. Lack of Standardized Monitoring Protocols

The clinical and toxicological data emerging from the included manuscripts show a significant variability across the studies. Reported analyses included blood, plasma, urine, vitreous humor, and cerebrospinal fluid (CSF), while techniques ranged from LC–MS/MS and HRMS to HPLC and immunoassays. However, the lack of consistent reference to alternative matrices and the limited number of available data points do not allow for an adequate pharmacokinetic assessment. This heterogeneity significantly hampers comparability between cases. Extracorporeal techniques, such as hemodialysis and continuous venovenous hemofiltration (CVVH), have demonstrated the ability to enhance baclofen clearance and reduce its half-life; however, no universally accepted guidelines exist regarding their indications, which limits their implementation in clinical practice.

In the context of intrathecal therapy, expert recommendations suggest a structured diagnostic approach to suspected pump malfunction, including prompt device interrogation, imaging of the catheter system, and supportive management of overdose with ventilatory support and, if necessary, CSF drainage [47]. However, delayed CSF removal may be ineffective in some cases, suggesting that early recognition and intervention are key determinants of outcome [48]. Mechanical failures- such as microleakage, disconnection, or catheter migration- can also produce abrupt clinical deterioration and should always be considered in the differential diagnosis of intrathecal overdose [49].

The implementation of evidence-based protocols is crucial to ensure the accurate monitoring and management of baclofen intoxications resulting from pump malfunctions. Moreover, such protocols would strengthen the forensic interpretation of cases where device failure is suspected [50].

### 4.4. Differentiation of Intoxication Contexts

The literature confirms that baclofen intoxications occur in heterogeneous scenarios. The Australian cohort showed that more than half of deaths were intentional overdoses, frequently in patients with psychiatric comorbidities and polysubstance use [23]. French data emphasized self-poisoning in the context of alcohol use disorder (AUD) [24]. Case reports have also documented accidental events, including intrathecal pump malfunctions, and recreational use among adolescents and young adults, sometimes with severe neurological presentations despite relatively modest plasma concentrations.

Our illustrative case falls within the iatrogenic/pump-related category, emphasizing the importance of tailoring preventive and diagnostic strategies to the specific context of intoxication. The increasing prescription of baclofen in alcohol-dependent patients with psychiatric comorbidities also emerges as a particular context of risk [50,51,52]. Franchitto et al. [53] reported a series of self-intoxications in this population, often involving co-ingestion of benzodiazepines and alcohol, with intentional overdose occurring at median doses around 340 mg. Although no fatalities were recorded, these findings highlight the importance of psychiatric screening and careful follow-up in high-risk individuals.

At a broader level, epidemiological surveys indicate that the misuse of prescription medications, including GABAergic agents such as baclofen, can reach prevalence rates comparable to those of traditional recreational drugs, emphasizing the need for awareness and prevention initiatives specifically addressing prescription misuse [54].

Distinguishing between therapeutic and recreational use is essential, as these patterns differ markedly in intent, risk profile, and clinical course, thereby influencing both preventive measures and the subsequent diagnostic and forensic approaches [55,56,57,58].

### 4.5. Postmortem Investigations

From a forensic perspective, only a limited number of studies provide detailed autopsy findings in baclofen fatalities [59].

None of the available reports present complete postmortem analyses or adequate descriptions of autopsy findings, and most rely solely on blood and urine testing. Furthermore, no investigations have specifically addressed postmortem redistribution or the influence of the postmortem interval, which further limits the interpretative reliability. Our case mirrored this limitation, as postmortem sampling was restricted to peripheral blood only; the absence of CSF and vitreous humor likely reduced interpretative confidence.

Nevertheless, systematic postmortem investigation is essential. Standardized collection of peripheral blood, vitreous humor, and CSF should be encouraged, together with the use of validated analytical methods such as LC–MS/MS or GC–MS, to improve the reliability and comparability of results, in determining the cause of death and to establish interpretative criteria.

High-resolution mass spectrometry (LC-QTOF-MS) has proven particularly useful in confirming baclofen intoxication in postmortem samples, allowing rapid and precise identification even at low concentrations [20]. Such techniques should be considered part of the modern forensic workflow for suspected baclofen-related deaths. Further research is required to establish the influence of postmortem interval on baclofen concentration in biological matrices due to intoxication [60].

Promoting systematic autopsy protocols and standardized diagnostic approaches, supported by multicenter data aggregation, would allow the collection of more robust and statistically meaningful evidence, improving both diagnostic accuracy and clinical management strategies in cases of suspected baclofen intoxication [61,62,63,64,65].

### 4.6. Future Perspectives

Further research is urgently needed to clarify the pharmacokinetics of baclofen, particularly in patients with renal impairment, in chronic high-dose users, and in cases of intrathecal administration. Prospective studies correlating blood and CSF concentrations with clinical manifestations would help to refine interpretation. Establishing multicenter registries and toxicological databases is crucial for expanding the caseload and facilitating the development of consensus guidelines. Shared endpoints should include matrix-specific reference ranges, PM interval-adjusted interpretation, and predefined thresholds for extracorporeal therapies. Given the increasing use of baclofen for AUD and its potential for misuse, continuous pharmacovigilance and toxicovigilance should be prioritized [34,35,48].

From a practical perspective, the implementation of shared clinical algorithms for intrathecal pump management and dose-based triage strategies for acute oral intoxications may represent achievable short-term objectives towards harmonizing clinical and forensic practice [47].

Overall, our illustrative case underscores and exemplifies the problems already evident in the literature: the inconsistency of toxicological thresholds, the variability of clinical presentations, and the medico-legal difficulties in postmortem interpretation. By integrating a case example with a systematic review, this study provides a structured framework for methodological proposals aimed at improving both clinical management and forensic toxicology practice.

## 5. Conclusions

Baclofen intoxication represents an emerging challenge in both clinical and forensic settings. The literature reveals substantial heterogeneity in presentation, toxicological findings, and outcomes, with fatal and non-fatal cases often overlapping in reported concentrations. Our illustrative case of intrathecal overdose exemplifies these interpretative difficulties, emphasizing the lack of standardized protocols for diagnosis, monitoring, and postmortem investigation.

The systematic review covering the past two decades confirms the scarcity of robust data and the absence of reliable cut-off values. To improve patient safety and medico-legal accuracy, there is an urgent need for multicenter registries, standardized toxicological panels, and consensus guidelines.

Future research should focus on pharmacokinetic characterization, prospective studies correlating concentrations with clinical severity, and clearly defined indications for extracorporeal treatments. By addressing these gaps, clinicians and forensic practitioners can develop more consistent and reliable strategies for managing baclofen intoxications.

## Figures and Tables

**Table 1:** Summary of the included studies in the review. All data reported were collected from previously published scientific articles and carefully verified by the authors for accuracy and consistency ([11,14,15,18,19,20,21,23,24,29,31,32]). Units of measurement are presented as reported in the original sources, and biological matrices are specified whenever available.

Year	Authors	Study Type	Sample (N)/Gender/Mean Age	Toxicological Method	Baclofen Analytical Data	Key Findings
2006	Shirley K.W. et al. [19]	Case Report	1; M; 12 years old	Not specified	7471 ng/mL (CSF)	Baclofen overdose and withdrawal are potentially life-threatening complications of pump and spinal catheter system malfunction
2012	Hsieh M.J. et al. [32]	Case Report	1; M; 55 years old	Not specified	1167 ng/mL (blood)	Patients with baclofen overdose could have prolonged elimination even with normal renal function
2012	Sauneuf B. et al. [18]	Case Report	1; M; 60 years old	High-performance liquid chromatography	2062 μg/mL (CSF)	Clinicians must be aware of the risk of intrathecal baclofen overdose and its acute management
2012	Sullivan R. et al. [14]	Brief Communication	2; 1 F and 1 M; 40 and 51 years old	Not specified	2.7 mcg/mL (blood; Not specified)	The need for more precise criteria to define brain death in intoxicated comatose patients
2015	Meulendijks D. et al. [21]	Case Report	1; M; 57 years old	LC-MS/MS method	1.81–0.05 mg/L (blood)	Continuous venovenous hemofiltration (CVVH)may be an alternative to hemodialysis in baclofen overdose
2015	Reichmuth P. et al. [31]	Case Report	1; M; 66 years old	Not specified	1510 mg/L (blood)	Off-label high-dose baclofen for a chronic alcohol use can precipitate severe toxicity, especially with renal dysfunction
2016	Szpot P. et al. [20]	Original Article	N = 1; F; 25 years old	High-Resolution Mass Spectrometry (HRMS)	30.7 μg/mL (blood)	A simple and rapid method for routine baclofen testing in autopsy blood
2017	Leger M. et al. [24]	Retrospective Study	190;~50% female, Median age 39–41 years	Not specified	Plasma baclofen concentrations were available for 30 patients with severe intoxications: median 3.24 mg/L compared to 0.32 mg/L in non-severe cases	Baclofen self-poisoning has increased with its use for alcohol use disorder
2020	Drevin G. et al. [11]	Review Article and Literature review	1; M; 16 years old; other not specified	Liquid chromatography-tandem mass spectrometry (LC–MS/MS) system	420 ng/mL in plasma and 64,900 ng/mL in urine; literature plasma <20–1322 ng/mL (non-fatal)	Psychotropic drug abuse is common among adolescents and young adults
2020	Farhat S. et al. [15]	Case Report and literature review of baclofen cases with EEG burst-suppression	1; M; 68 years old	Not specified	4.30 ug/mL (blood)	Severe baclofen toxicity can mimic brain death; EEG can help avoid premature prognosis
2020	Khatun Nahar L. et al. [22]	Original Article	21; 11 male (52%) and 10 female (48%); the age ranged from 31 to 69 years (median = 47 years) for male and 31 to 71 years (median = 52 years) for female	Liquid chromatography–tandem mass spectrometry (Triple Quadrupole mass spectrometer)	from 0.08 to 102.00 μg/mL (median = 0.28 μg/mL, mean = 5.90 μg/mL ± 22.2 SD) (femoral vein blood)	This study highlights that although Baclofen abuse may be occurring, deaths are rare
2024	Dudek C.M. et al. [29]	Case Report	1; F; 70 years old	Gas chromatography-mass spectrometry and high-performance liquid chromatography (LCMS), high- resolution mass spectrometry (LC-HRMS)	4.4 mcg/mL (blood; Not specified)	Baclofen overdose can feature cardiovascular complications (ECG changes/Takotsubo pattern)
2024	Zahra E. et al. [23]	Retrospective Study	102; 51% being male; mean age of 45.6 years	Immuno- assay, gas chromatography, high-performance liquid chromatography (HPLC) and liquid chromatography-quadrupole time-of-flight mass spectrometry (LC-QTOF-MS	The median baclofen blood concentration for all cases was 3.10 mg/L (25th 0.70, 75th 8.10, range 0.04–110.00), unintentional toxicity 1.95 mg/L (25th 0.70, 75th 4.35, range 0.04–24.0), intentional toxicity 6.00 mg/L (25th 1.10, 75th 13.0, range 0.05–110.0)	Caution is needed in prescribing baclofen given its potential to be used in intentional and non-intentional overdose

## Data Availability

The data presented in this study are available on request from the corresponding authors.
